# Supplementary material for: Genome-Wide Association Studies of Estimated Fatty Acid Desaturase Activity in Serum and Adipose Tissue in Elderly Individuals: Associations with Insulin Sensitivity
Source: Nutrients. 2018 Nov 17;10(11):1791. doi: 10.3390/nu10111791 (PMC6266021; doi:10.3390/nu10111791)
Supplement: Supplementary file 1 [file nutrients-10-01791-s001.pdf]

Supplementary material

Genome-Wide Association Studies of Estimated Fatty Acid Desaturase Activity in Serum and Adipose Tissue in Elderly Individuals: Associations with Insulin Sensitivity

Matti Marklund, Andrew Morris, Anubha Mahajan, Erik Ingelsson, Cecilia Lindgren, Lars Lind and Ulf Risérus

Contents

Supplementary Figures 2

Figure S1. Manhattan plot of the meta-analysis of the single-variant association results of serum D5D. 2

Figure S2. Manhattan plot of the meta-analysis of the single-variant association results of serum D6D. 3

Figure S3. Manhattan plot of the meta-analysis of the single-variant association results of serum SCD. 4

Supplementary Tables 5

Table S1. Sample exclusion criteria and number of samples excluded after quality control in PIVUS and ULSAM. 5

Table S2. Secondary signals significant at P-value ~10^{-5} after adjustment for the lead variant. 6

Table S3. Results for the significant loci in the single variant analysis extracted from published meta-analyses of GWAS for HOMA-IR, HDL cholesterol, triglycerides, and BMI. 7

Supplementary Figures

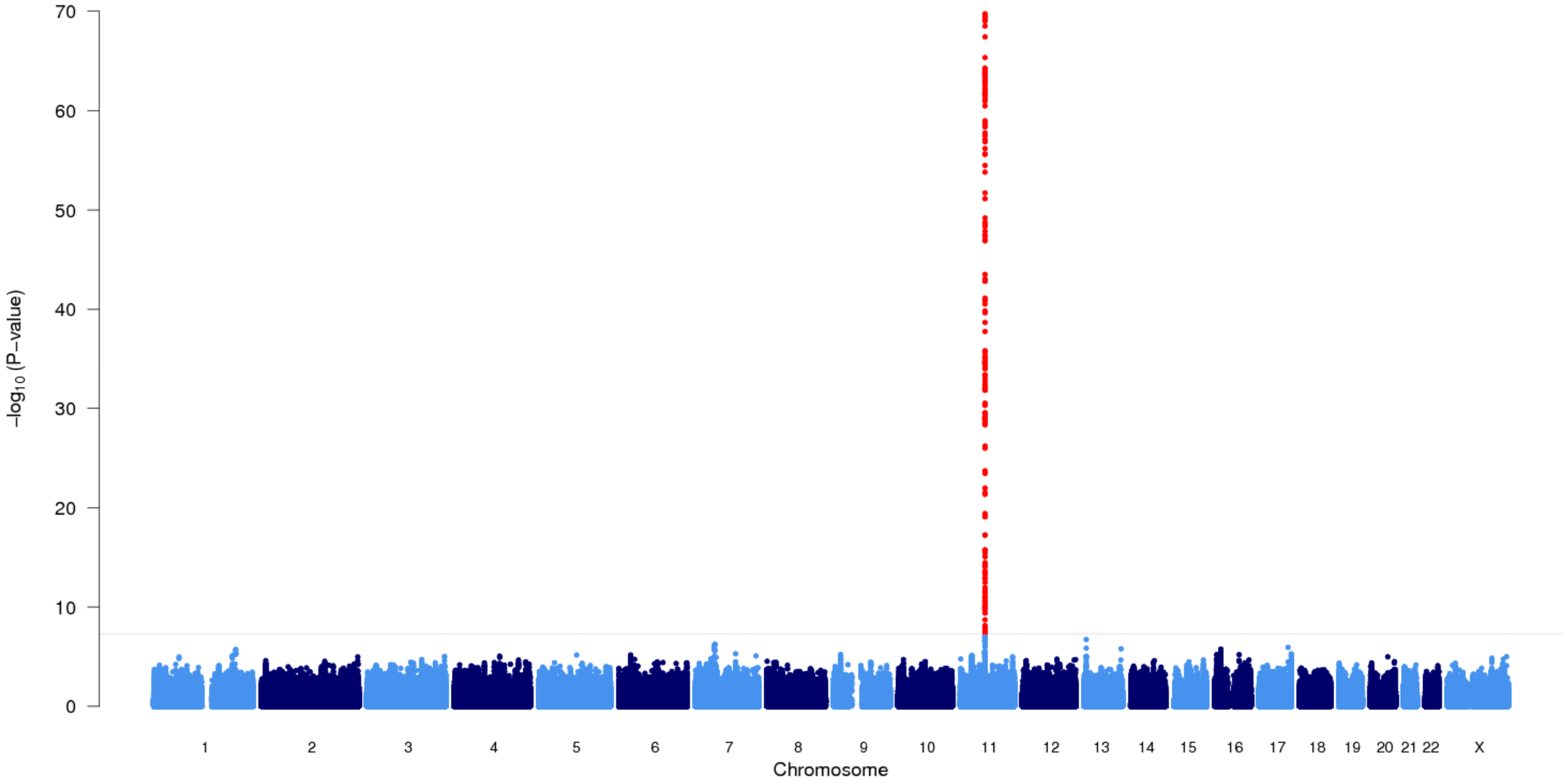

Figure S1. Manhattan plot of the meta-analysis of the single-variant association results of serum D5D.

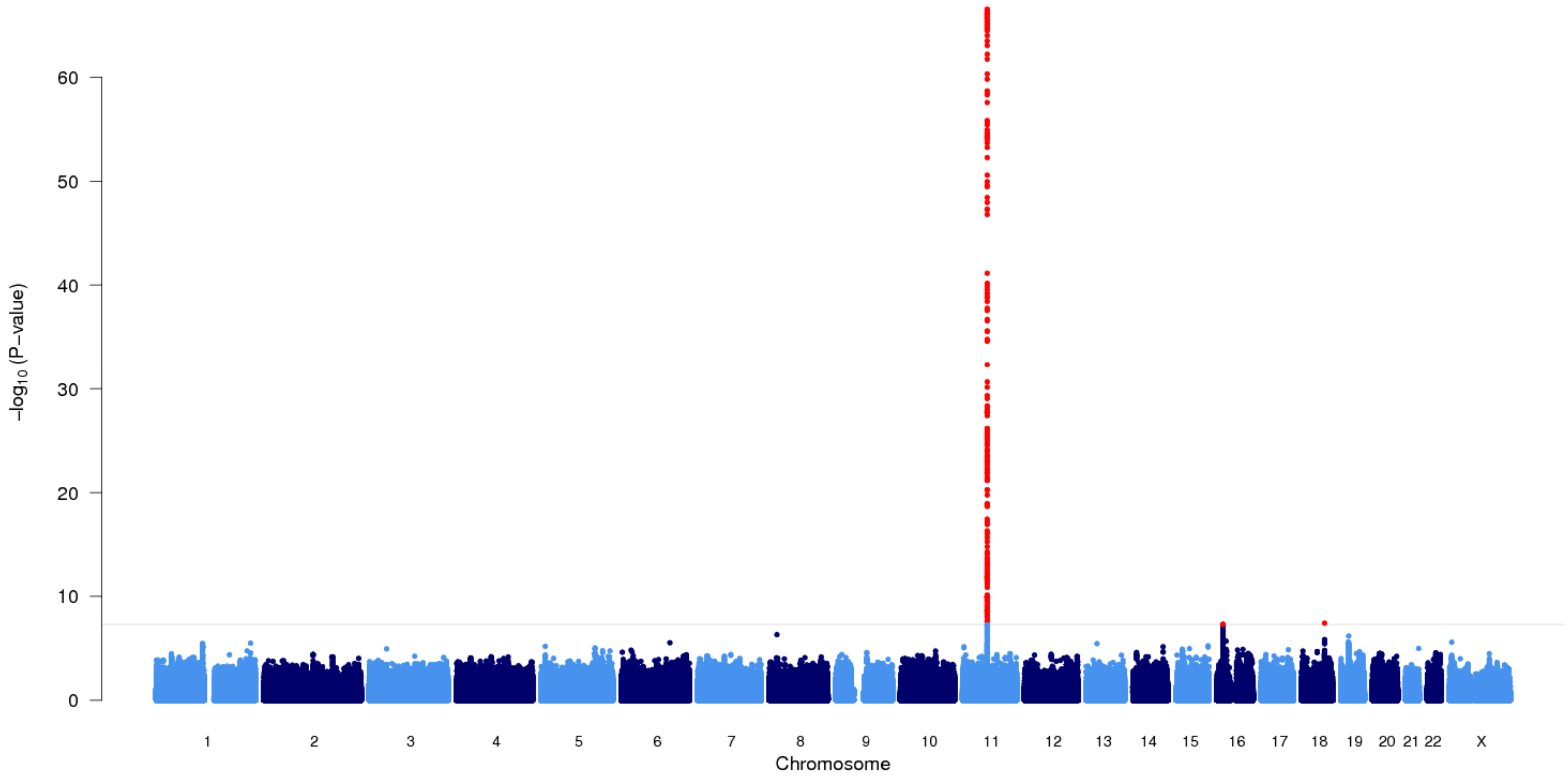

Figure S2. Manhattan plot of the meta-analysis of the single-variant association results of serum D6D.

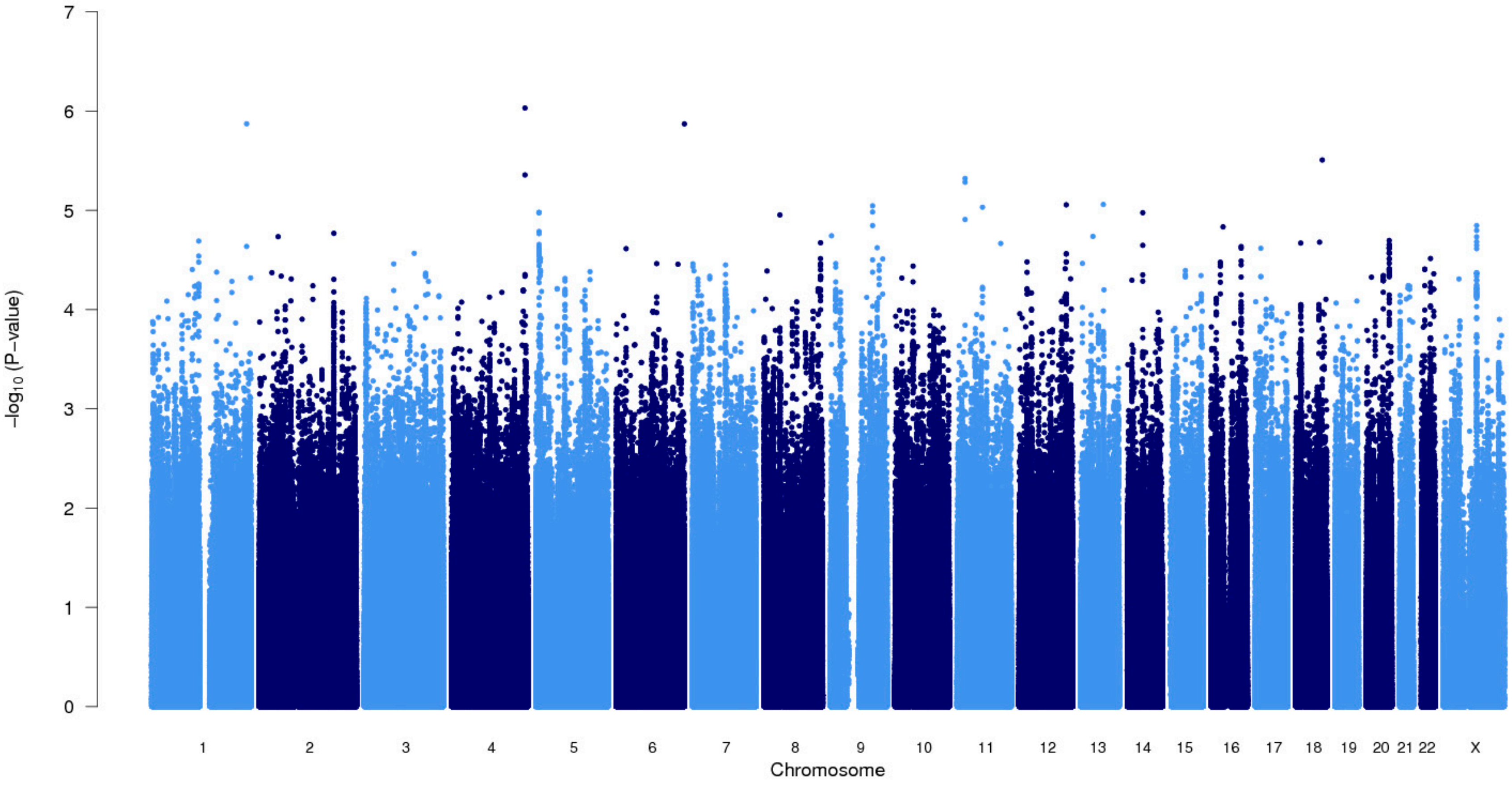

Figure S3. Manhattan plot of the meta-analysis of the single-variant association results of serum SCD.

Supplementary Tables

Table S1. Sample exclusion criteria and number of samples excluded after quality control in PIVUS and ULSAM.[a]

		Sample exclusion criteria					
Cohort	Genotyping platform	Call rate<95%	Heterozygosity >3 SD	Gender discordance	Duplicates	IBD match	Ethnic outliers
PIVUS	Illumina OmniExpress	7	14	3	0	0	0
	Illumina Metabochip	0	6	0	3	0	0
ULSAM	Illumina OmniExpress	6	7	1	0	1	22
	Illumina Metabochip	0	0	0	0	0	0

[a]IBD, identity by descent; PIVUS, Prospective Investigation of Uppsala Seniors; SD, standard deviation; ULSAM, Uppsala Longitudinal Study of Adult Men.

Table S2. Secondary signals significant at P-value ~10^{-5} after adjustment for the lead variant.[a]

					Effect allele /	Before adjustment		After adjustment	
Desaturase	lead variant	rs#	Chr:Position (b37)	EAF	Other allele	Direction	P-value	Direction	P-value
D5D	rs174549	rs968567	11:61595564	0.15	T / C	-	4.8×10^{-49}	-	1.7×10^{-5}
D6D	rs138194593	rs2072113	11:61604967	0.17	T / C	-	3.8×10^{-55}	-	3.3×10^{-9}

[a]b37, NCBI build 37; Chr, chromosome; D5D, δ-5-desaturase; D6D, δ-6-desaturase; EAF, effect allele frequency; FADS, fatty acid desaturase.

Table S3. Results for the significant loci in the single variant analysis extracted from published meta-analyses of GWAS for HOMA-IR, HDL cholesterol, triglycerides, and BMI.[a]

		Effect		HDL[b]			Triglycerides[b]			BMI[c]			HOMA-IR[d]		
Gene	rs#	Allele	EAF	Direction	P-value	N	Direction	P-value	N	Direction	P-value	N	Direction	P-value	N
FADS1	rs174549	a	0.32	-	4.3×10^{-23}	186973	+	6.9×10^{-34}	177673	+	0.47	321655	+	0.60	46186
FADS2	rs968567	t	0.16	-	6.2×10^{-04}	172673	+	3.4×10^{-09}	163958	+	0.40	309670	+	0.14	46186
FADS2	rs2072113	c	0.85	+	9.1×10^{-05}	90617	-	1.0×10^{-06}	84618	N/A	N/A	N/A	N/A	N/A	N/A
PDXDC1	rs6498540	a	0.67	+	0.03	92820	-	9.8×10^{-04}	89485	+	1.3×10^{-03}	233998	+	0.55	46186
near MC4R	rs9957425	c	0.39	-	0.24	92820	+	0.04	89485	+	0.55	220175	-	1.00	46186
PKD2L1	rs603424	a	0.19	+	0.52	187113	+	0.49	177807	-	0.73	309733	+	0.28	46186

[a]BMI, body mass index; EAF, Effect allele frequency; HDL, high-density lipoprotein; HOMA-IR, homeostasis model assessment of insulin resistance. [b]Results from (PubMed ID: 24097068). [c]Results from (PubMed ID: 25673413). [d]Results from (PubMed ID: 20081858).
